# Does competition improve hospital performance: a DEA based evaluation from the Netherlands

**DOI:** 10.1007/s10198-022-01529-8

**Published:** 2022-10-04

**Authors:** Peter Dohmen, Martin van Ineveld, Aniek Markus, Liana van der Hagen, Joris van de Klundert

**Affiliations:** 1 Rotterdam School of Management, Erasmus University, Rotterdam, The Netherlands; 2 Erasmus School of Health Policy and Management, Erasmus University, Rotterdam, The Netherlands; 3 Department of Medical Informatics, Erasmus University Medical Center, Rotterdam, The Netherlands; 4 School of Business, Universidad Adolfo Ibanez, Santiago de Chile, Chile; 5 Dutch Healthcare Authority (Nederlandse Zorgautoriteit; NZa), Utrecht, The Netherlands

**Keywords:** Data envelopment analysis, Hospital performance, Productivity, Competition in healthcare

## Abstract

Many countries have introduced competition among hospitals aiming to improve their performance. We evaluate the introduction of competition among hospitals in the Netherlands over the years 2008–2015. The analysis is based on a unique longitudinal data set covering all Dutch hospitals and health insurers, as well as demographic and geographic data. We measure hospital performance using Data Envelopment Analysis and distinguish three components of competition: the fraction of freely negotiated services, market power of hospitals, and insurer bargaining power. We present new methods to define variables for each of these components which are more accurate than previously developed measures. In a multivariate regression analysis, the variables explain more than half of the variance in hospital efficiency. The results indicate that competition between hospitals and the relative fraction of freely negotiable health services are positively related to hospital efficiency. At the same time, the policy measure to steadily increase the fraction of health services contracted in competition may well have resulted in a decrease in hospital efficiency. The models show no significant association between insurer bargaining power and hospital efficiency. Altogether, the results offer little evidence that the introduction of competition for hospital care in the Netherlands has been effective.

## Introduction

Hospitals accounted for 39% of total health expenditures in OECD countries in 2019 on average [57]. Because of this large share and perceived inefficiencies in their operations, hospitals continue to be placed at the centre of cost containment policies by governments. To improve hospital productivity, many governments have introduced policies introducing various forms of competition—which might ‘save lives without raising costs’ [31] p.134)—in combination with supply-side regulations [18, 63]. In theory, the introduction of properly managed competition should yield the desired hospital productivity, as patients and payers will be more likely to choose better-performing hospitals [25]. On the other hand, it has been argued that market flaws such as information asymmetry, lack of transparency, and complexity cause competition to be ineffective for healthcare markets [61, 46]. Whether managed competition in healthcare is helpful or harmful, good or bad, is very much an open question still, and subject to ongoing debate [6, 22, 32].

Managed competition was introduced in the Netherlands in 2006, gradually implemented over subsequent years, and incrementally adjusted based on evaluation findings. The effectiveness of the reform continues to be disputed [24, 39, 44, 68]. In this study we advance the evidence base on the effectiveness of managed competition in the Netherlands, analysing hospital productivity over the period 2008–2015, in relation to measures of competition. This analysis is based on a unique data set spanning eight years of nationwide financial data from hospitals and insurers, as well as demographic and geographic data. Moreover, we present new methods to develop variables that capture the elements of managed competition.

Previous research on the effects of competition on hospital performance is equivocal. Melnick et al. [49] reported that hospital competition caused price reductions in the US, a finding confirmed by later studies [73, 83]. Research from the US on hospitals in highly concentrated provider markets shows that insurers have the bargaining power to reduce hospital prices [65]. In the UK, where single-payer patient-driven competition was introduced based on fixed prices, Propper et al. [62] found a negative relationship between competition and quality of care. However, Cooper et al. [18] found that mortality, as a proxy for quality, reduced after the implementation of market reforms in the UK. Studies focusing specifically on hospital efficiency also paint a mixed picture, suggesting that national contexts and reform designs play an important role. Lee et al. [40] found that for US hospitals efficiency increased with more intense competition. Positive relationships were also found between competition and the efficiency of public hospitals in Australia [16] and teaching hospitals in the US [34]. Research in the UK showed ambiguous results [42]. By contrast, Narcı et al. [52] showed the degree of competition among hospitals was not related to hospital efficiency in Turkey. In Germany and Italy negative relationships between components of competition and hospital efficiency were found [17, 36]. In Taiwan, Chu et al. [15] found no relationship between market competition and hospital efficiency.

The introduction of managed competition in the Netherlands in 2006 was founded in the pre-existing multi-payer system while introducing mandatory health insurance. In the new system, insurers, patients, and hospitals form a triadic relationship [79] and there is managed competition in three markets: patient-driven competition in the market between patients and hospitals [39]; payer driven competition in the market between insurers and hospitals [66, 77], and the third market between patients and insurers [44, 79]. Dutch patients are given a free choice of health insurer and the health insurers are expected to act prudently on behalf of the patients when contracting with providers. The design envisions insurers to promote access, efficiency, and quality of hospital care, in alignment with patient preferences [2, 44, 76]. For this purpose, insurers can negotiate volume, price, and quality for a considerable set of health services and can contract selectively. The gradual implementation is for instance illustrated by the stepwise increase in the volume of services for which prices, volume and quality were freely negotiable—from approximately 10% of the hospital budget in 2006, to 20% in 2008, 34% in 2009 and 70% in 2012 [53]—and by the considerable adjustment of the DTC (Diagnosis Treatment Combination) payment system, the Dutch variation of DRG, in 2012 [24, 49, 54].

As there are no counterfactual (a control Netherlands without regulated competition) robust research designs to answer questions about the attribution of (changes in) hospital performance to the reform are hardly available. However, because of the gradual implementation and adjustment of the various components of managed competition introduced, a longitudinal approach towards evaluation of the effects of managed competition can robustly assess the relationship between the reform and hospital performance. Following this approach, our research investigates the association between components of managed competition and the productivity of Dutch hospitals in the period 2008–2015. It will do so on the basis of a comprehensive data set which includes data from all Dutch hospitals and health insurers over this eight years period. The longitudinal approach allows to answer two research questions. Adopting the view that poor relative performance will be less feasible as competition increases, the first question evaluates whether managed competition is associated with the productivity of a hospital compared to other hospitals (relative efficiency). Likewise, one may expect that gradual increases in the competition are associated with year-to-year improvements in relative efficiency (technical efficiency), as assessed in a second research question.

## Modeling the relationship between managed competition and hospital performance

**Fig. 1:**
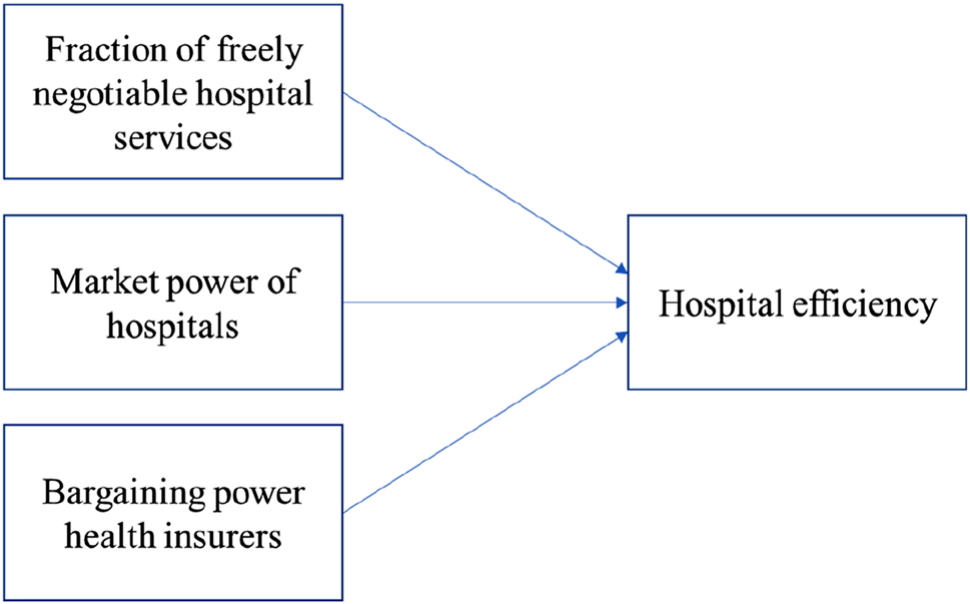
The relationships between managed competition components and hospital performance

The Dutch health system design assumes health insurers leverage their market position and bargaining power to negotiate contracts with hospitals that foster hospital efficiency. The ability of health insurers to achieve efficiency improvement is influenced by various characteristics of the markets between health insurers and hospitals, and patients and hospitals [7]. In our model, we distinguish three components of managed competition that relate to hospital efficiency: (1) the fraction of freely negotiable hospital services, (2) market power of hospitals in the patient-driven competition and (3) bargaining power of health insurers in the payer-driven competition. Figure 1 visualizes these relationships. Each component is included in a regression model, using one or several independent variables to explain the dependent variable hospital efficiency. Before giving the model specifications, we provide an in-depth discussion of the variables operationalizing the components.

The first component regards the fraction of freely negotiable hospital services for which health insurers and hospitals negotiate on price, volume and quality. In practice, this fraction is primarily viewed as a nationwide reform measure, defined as the revenue from health services for which prices are freely negotiable divided by total hospital revenue from health services. This financial operationalization follows the current contracting practice between Dutch health insurers and hospitals which are dominated by price negotiations [71]. As mentioned in the introduction, the fraction of freely negotiable prices increased gradually as the Dutch managed competition design assumed that competition will cause hospital efficiency to improve when the fraction of freely negotiable prices increases [77]. It should be noted that previous evidence suggests the opposite may have happened [78].

The second component in our conceptual model is the market power of hospitals on the market between patients and hospitals. This market of patient-driven competition has received attention in a variety of countries for its possible relationship with hospital performance [11, 16, 42, 45]. In particular, there is substantial evidence of prices being higher when there are relatively fewer hospitals (which therefore might have more power) [14, 20, 27, 30, 41, 74]. A recent study by Gajadien et al. [29] shows that hospitals with a higher market share are able to include lower financial risks in their contracts with health insurers.

The third component regards the bargaining power of insurers on the market between insurers and hospitals. There is evidence on payer-driven competition stating that health insurers more effectively influence the efficiency of a hospital as their share in the total volume of services contracted at that hospital is larger [50, 51, 74]. It should be noted, however, that Van Dijk et al. [75] found no relation between health insurer competition and prices of disease management programs in Dutch primary care. Schut and Varkevisser [67] argue that the bargaining position of Dutch health insurers is still relatively weak in general.

## Methods

### Dependent variable: hospital efficiency

The dependent variable of hospital efficiency is operationalized using data envelopment analysis (DEA). DEA is widely used in healthcare research and has proven to be an effective tool for analysing the relative efficiency of hospitals and the impact of health system reforms on hospital’s efficiency [38, 47]. The relative efficiency addressed in the first research question is captured through the DEA scores of hospitals per year. We let the variable DEA(*h*, *t*) reflect the DEA efficiency score of hospital *h* in year *t*. The technical efficiency studied in the second research question is obtained from decomposing the corresponding Malmquist Indices (MI), as addressed below.

Since the main objective of the introduction of managed competition was to encourage hospitals to deliver the required outputs with as few inputs as possible, we adopted an input-oriented DEA model. Next, we motivate our choice of a Constant Returns to Scale (CRS) model as a default model. Firstly, we note that all Dutch general hospitals have at least 200 beds and previous studies [10, 78] have consistently concluded that scale effects appear to be absent or negative. Hence, there is no need to cope with hospitals in catchment areas with population sizes that are too small to reach competitive economies of scale. We, therefore, view scale as a strategic choice of the hospitals, which are private entities, and as one of the decision variables available to hospital management to improve efficiency. This approach is consistent with the policy reforms and the introduction of managed competition in which scale is not explicitly considered. The alternative of a Variable Returns to Scale (VRS) model would blur scale-related inefficiencies and complicate answering the research questions. As a form of sensitivity analysis, the results of a Variable Returns to Scale (VRS) model are nevertheless presented in Appendix 1.

The MI is often preferred to analyse the efficacy of health system reform over time using panel data and instrumental in answering the second research question which regards technical efficiency improvements between 2008 and 2015 [38]. For hospital *h* in year *t*, variable MI(*h*, *t*) expresses the change in relative hospital efficiency from year *t* to year *t* + 1. MI(*h*, *t*) can be decomposed into a technological change (frontier-shift effect), which measures the change of the efficiency border, and a technical efficiency change (catch-up effect), which evaluates the change in the efficiency of a hospital between two consecutive years [26]. As the technological changes relevant for hospitals over the period 2008–2015 are largely exogenous and, therefore, not causally related to the system reforms, the second research question is answered using the technical efficiency changes, which capture whether hospital performances move closer to the efficiency frontier over time.

The input and output variables considered in the DEA model are presented below. Table 1 presents a descriptive overview of all measures for the selected hospitals. MI uses the same input and output variables. DEA assumes an isotonic relation between inputs and outputs, i.e., the value of each of the inputs is positively associated with the value of each of the outputs [33]. We indeed find that the correlation coefficients between all selected input and output variables are positive (see Appendix 7, Table 14).

**Table 1:** Descriptive statistics of Dutch hospital variables (2008–2015) included in the DEA/MI (*N* = 576)

Variable	Mean	Std. dev	Min	Max
Input				
FTE (total, incl. physicians)	1.758	931	456	4911
Operating expenses	€58.133.348	€35.667.206	€12.362.298	€200.237.003
Output				
Number of admissions	21.221	9.441	5.965	47.423
Number of daycare treatments	21.897	10.429	4.858	58.367
First outpatient visits	122.000	51.483	36.392	256.000

#### DEA input measures

DEA models typically consider capital, labour, and operating expenses as the main inputs [38, 58, 59]. Accounting for around 60% of the total annual costs, labour can be considered as the most important input of Dutch hospitals [9]. We specified labour in full-time equivalents (FTE)—rather than cost—and more specifically as the total number of medical doctors, other medical personnel and non-medical personnel working in the hospital. Operating costs (other than expenses for labour) form around 30% of annual hospital costs, which were extracted directly from the annual reports. This literature-based selection of input variables represents mutually exclusive inputs and cover more than 90% of all inputs of Dutch hospitals in financial terms [9]. These data are available from the mandatory, audited, annual reports issued by Dutch hospitals.

Lacking robust financial data, many DEA studies specify capital investment in terms of a number of beds. In the Netherlands, the reported number of beds often deviates from the actual number of beds in operation. Dutch hospitals, however, present audited financial data annually, including a balance sheet. Unfortunately, the financial reporting standards for the annual reports have changed considerably and multiple times over the period of study, causing inconsistencies and considerable adjustments in the balance sheet asset valuations over the years. As a consequence, they are unreliable as a measure of the production factor capital on a yearly basis [78]. Ultimately, we decide not to include the input variable capital, which accounts for around 6% of total costs for Dutch hospitals [9].

#### DEA output measures

In most studies, inpatient and outpatient data are used as output categories [38]. We distinguish between the number of (inpatient) admissions, number of primary outpatient visits, and the number of day care treatments as the three output measures in our DEA model. The selected output measures are the three output indicators available at the hospital level in the Netherlands, which are mutually exclusive and together fully cover the production of hospitals for insured care [23]. These three variables are consistently reported since the start of the reform in 2006 in the mandatory, audited, annual reports.

### Independent variables

The components of managed competition, i.e. freely negotiable prices, market power of hospitals, and market power of health insurers are operationalized into five independent variables as follows.

The first two variables regard the fraction of freely negotiable health services. The set of health services to which free negotiations apply is called the B-segment (the A-segment forming the set of services for which prices and volumes are non-negotiable). The foundational measure for the first component is therefore the fraction of total hospital revenue earned from revenue from B segment services. Let ***Η*** = {1, …, *H*} be the set of all hospitals. For *h* = 1,… *H*, and *t* = 2008, …, 2015, let *b*(*h*, *t*) be the monetary value of freely negotiable hospital services for hospital *h* in year *t*, and let *r*(*h*, *t*) be the total hospital revenue from insured care for hospital *h* in year *t*. Next, for *h* = 1, …, *H*, and *t* = 2008, …, 2015), we define

$$B\left(h,t\right)=\frac{b(h,t)}{r(h,t)}.$$

The values *B*(*h*, *t*) differ among hospitals and over the years because of arrangements made between health insurers and hospitals and the resulting health services actually provided. For instance, hospitals focusing on standardized health services in the B-segment typically have a larger *B*(*h*, *t*) whereas teaching hospitals typically have a lower *B*(*h*,* t*). Over the years, these cross-sectional differences among the hospitals may change. At the same time, substantial increments in the *B*(*h*, *t*) have occurred over the years because of the gradual implementation of the nationwide reform. To distinguish this longitudinal effect from the cross-sectional effect, we define

$$B\left(t\right)= \frac{{\sum }_{h=1}^{H}b(h,t)}{{\sum }_{h=1}^{H}r(h,t)} \quad \mathrm{for} \quad t = 2008,\ldots ,2015,$$

which is the yearly nationwide fraction of hospital revenue earned from freely negotiated hospital services. Hence, *B*(*t*) is a yearly fraction for all hospitals together. For the hospitals included in our dataset, the fraction of freely negotiated hospital services has incrementally increased from *B*(2008) = 0.24 to *B*(2012) = 0.93. We use independent variable *B*(*t*) to analyse the longitudinal effect.

To be able to distinguish the hospital-specific, cross sectional, variations in *B*(*h*, *t*) from the nationwide increments *B*(*t*), we define

$$\Delta B\left(h,t\right)=B\left(h,t\right)-B\left(t\right) \quad \mathrm{for } \quad h=1,\dots ,H \; \mathrm{ and } \; t = 2008,\ldots ,2015.$$

The Δ*B*(*h*, *t*) variables capture for each hospital *h* and in year *t* the fraction of freely negotiated health services, relative to the national weighted average in year *t*. One might hypothesize that more efficient hospitals offer lower prices and grow their market share for services with freely negotiable prices, resulting in a positive Δ*B*(*h*, *t*), counterbalanced by negative Δ*B*(*h*, *t*) for less efficient hospitals. Alternatively, one might view that hospitals with larger Δ*B*(*h*, *t*) are more exposed to market forces and therefore need to be more efficient to be sustainable. We will address possible dependency of the Δ*B*(*h*, *t*) on the DEA(*h*, *t*) by inspecting the results of a linear regression model in which the Malmquist Index scores MI(*h*, *t*) replace the DEA(*h*, *t*) as a dependent variable.

Teaching and research activities obviously impact the financials and the efficiency of hospitals, and hospitals that teach and conduct research also typically serve a patient population with a higher case load. The eight tertiary, academic hospitals have a distinct position in the Netherlands and have essentially different cost and revenue bases. Hence, we have excluded those from the study. The Dutch health system does not explicitly distinguish teaching hospitals, nor does it provide other uniformly reported measures capturing case mix. However, general hospitals which provide more advanced services have united themselves in an association called STZ. STZ-affiliated hospitals tend to provide less services in the freely negotiated B segment. We explicitly model STZ affiliation by binary variable STZ(*h*), *h* = 1,…, *H* which is 1 for STZ members and 0 for others. This variable controls for teaching status and case mix.

For the second component, we consider the operationalization of the market power of hospitals on the market of patients selecting hospitals services. Varkevisser et al. [80] find that Dutch patients allow a maximum travel distance of 30 min and that within this limit, hospitals are considered less attractive as the travel time increases. Roos et al. (2020) present a model to capture the market share of Dutch hospital in the market of patients on the basis of the 30 min maximum, without accounting for proximity within this limit of 30 min. Moreover, their model uses distances between hospitals instead of travel times of patients and relies on the number of beds as a proxy for hospital size (which is not accurate as discussed above). We now present an alternative measure below that remedies these three shortcomings.

Intuitively, the market power of hospital *h* is considered high if patients living within 30 min travel distance have no alternative hospital within travel distance. The market power of a hospital decreases as the number of patients within this travel distance for which there is an alternative hospital within travel distance increases, and as this number of alternative hospitals travel distance increases.

Now formally, we define for all persons *p* of the Dutch population *P*, and for all hospitals *h*,* h* = 1, …, *H*, the travel time *t*(*h*, *p*). Moreover, for all members *p* of the Dutch population *P*, and for all hospitals *h*, *h* = 1,…, *H*, we define the reachability of hospital *h* for person *p* by

$$r\left(h,p\right)=\mathrm{max}\left(0, \frac{30-t\left(h,p\right)}{30}\right).$$

Thus, the reachability is 1 if the travel time distance is 0, and decreases proportional to the travel time, becoming 0 for all travel times of 30 min and above. Parameter *R*(*h*, *p*) then defines whether hospital *h* is reachable for person *p*, i.e.,* R*(*h*,*p*) = 1 if *r*(*h*, *p*) > 0 and 0 otherwise.

Based on these parameters, we define for all members *p* of the Dutch population *P*, and for all hospitals *h*, *h* = 1, …, *H*, the attractiveness $a(h,p)$ by setting

$$a\left(h,p\right)= \frac{r\left(h,p\right)}{\sum_{j=1}^{H}r\left(j,p\right)}.$$

The intuition behind the attractiveness is as follows. For any person *p*, hospital *h* has an attractiveness of 1 if it is the only hospital with travel distance, i.e. for which *r*(*h*, *p*) > 0. For a person *p* having multiple hospitals *h* with *r*(*h*, *p*) > 0, the *a*(*h*, *p*)’s are proportional to the *r*(*h*, *p*)’s and add up to 1 (i.e., they are normalized). One interpretation is that the *a*(*h*, *p*)’s are the probabilities for hospital *h* to be selected by person *p*, when assuming that these probabilities are linearly decreasing with travel times, up to a travel time of 30 min.

With these notations at hand, we finally define the attractiveness *A*(*h*) of each hospital *h*, *h* = 1, …, *H*:

$$A\left(h\right)= \frac{\sum_{p\in P}a(h,p).}{\sum_{p\in P}R(h,p)}.$$

Thus, the attractiveness of hospital *h* equals the average attractiveness of *h*, over the patients for which it is reachable (within 30 min travel time). This hospital’s attractiveness will be our measure of hospital market power. For hospitals reachable by patients with few (nearby) alternatives, the attractiveness scores will be high. For a monopolist, i.e., a hospital that is only reachable by patients for which all other hospitals are at least 30 min away, the attractiveness equals 1. It decreases as patients who can reach a hospital have more (nearby) alternatives.

The third component regards insurer market share in the market of purchasing hospital services. Let {*1*,…, *I*} be the set of all insurers, and let *b*(*i*, *h*, *t*) be the monetary value of freely negotiable hospital services contracted by insurer *i* from hospital *h* in year *t*, for *i* = 1, …, *I*, *h* = 1, …, *H*, *t* = 2008, …, 2015. Then we define the annual contracting volume of insurer *i* at hospital *h* in year *t* as

$$\mathrm{IMS}\left(i,h,t\right)= \frac{b(i,h,t)}{b(h,t)} \quad \mathrm{for } \quad i=1,\dots ,I, h\hspace{0.17em}=\hspace{0.17em}1,\dots ,H, t\hspace{0.17em}=\hspace{0.17em}2008,\dots ,2015.$$

In the analysis, we consider various independent variables defined on the basis of IMS(*i*, *h*, *t*). As mentioned above, the annual contracting volume determines the bargaining power of health insurers and therefore its capability to impact hospital efficiency. Insurers with a smaller annual contracting volume are likely to be less impactful than insurers with larger annual contracting volumes. Likewise, if many health insurers all have equal purchasing volumes at a certain hospital, it is difficult for any of them to have a distinguishable impact on 6hospital efficiency. Insurers are not allowed to overcome this difficulty by collaboration, as the regulations stipulated to safeguard competition severely limit alignment between insurers.

Reasoning along these lines we hypothesize that the effects of insurer purchasing practices on hospital efficiency are more profound when one or a few insurers have considerably larger volumes and we present various variables and models to capture such effects. Before presenting a classic model based on the Herfindahl–Hirschmann Index [35], we firstly present models that consider the purchasing volumes of the largest and second-largest insure per hospital, following current practices in the Netherlands in which these two insurers play a representing role.

For hospital *h* and year *t*, let IMS(*h*, *t*)* and IMS(*h*,*t*)** be the largest and second largest annual contracting volumes among all health insurers *i* (breaking ties arbitrarily) and let i* be any insurer such that IMS(i*, *h*, *t*) = IMS(*h*, *t*)*, for *i* = 1,… *I*, *h* = 1, … *H*,* t* = 2008, …, 2015. Then, two first operationalizations of insurer market share are:

- max(IMS(*h*,*t*)* – 0.5, 0), i.e. the largest insurer market share minus 0.5, provided it is larger than the total of the other market shares. This caters for instance for the situation where one insurer contracts 80% of the B-segment services at a certain hospital.
- IMS(*h*, *t*)* and IMS(*h*,t)**, provided that the second largest insurer has a market share which is larger than the sum of the market shares of the number three onwards, i.e. IMS(*h*, *t*)** ≥ 1—IMS(*h*, *t*)*—IMS(*h*, *t*)**. This caters for instance to the situation where two insurers each contract about 40% of the B-segment services at a certain hospital.
  These operationalizations disregard the relative effectiveness of insurer practices. Some insurers may be more effective at translating their purchasing power into hospital efficiency than others. Hence, we also analyse an independent variable expressing whether insurer *i* procures more than half of the B segment volume at hospital *h* in a certain year *t*:
- IL(*i*, *h*, *t*)* = 1 if *i* = i* and IMS(*h*, *t*)* ≥ 0.5, 0 otherwise, for *i* = *1*,…,*I*, *h* = *1*,… *H*, *t* = 2008, …, 2015.
  The fourth variable to operationalize purchasing power is a commonly used measure for concentration: the Herfindahl–Hirschman Index (for closely related applications see [35, 74], which is defined as:
- HHI (*h*, *t*) = $\sum_{i=1}^{I}$IMS (*i*, *h*, *t*)^2^ for *i* = 1,…, *I*, h=1,…*H*, *t* = 2008,…,2015.

This index has the smallest value when all insurers have equal market shares and equals 1 if some insurer purchases all services at hospital *h* in year *t*. We consider HHI to be our key variable measuring health insurer’s bargaining power.

### Regression models

The lower and upper bounds of 0 and 1 of an input-oriented DEA model have led various researchers to adopt different linear regression models with DEA scores as the dependent variable. McDonald [48] points out that the DEA score bounds are not due to truncation or censoring of data, and he, therefore, argues for an ordinary least squares (OLS) model. However, as DEA scores are skewed, using OLS could lead to inappropriate estimations [43]. A log-odds transformation (logit) of the dependent DEA variable can solve this problem [12].

Simar and Wilson [69] argue that DEA scores result from a stochastic process as they are calculated from a sample of observations and measurement errors may occur for each of the input and output parameters. To better accommodate the stochastic nature of the data, they developed a two-stage estimation bootstrap procedure. In pursuit of robustness, this study reports results obtained from all three approaches as motivated and detailed below.

The data set considered in this study considers the complete set of Dutch hospitals and the included data are taken from mandatory yearly reports which have obtained accountant approval. This limits the validity of some of the motivations to prefer a stochastic frontier approach. As argued by McDonald [48], the application of traditional DEA methods to the complete and accurate set of actual empirical data at hand may better suit the evaluative nature of our study. Thus, we first applied OLS regression models to estimate the associations of the variables representing the components of managed competition with the dependent variable DEA score. This analysis starts from univariate regression models for each of the components presented above and then considers multivariate models in which all components are considered jointly.

In the final three multivariate models, we use four variables for the bargaining power of health insurers. To avoid possible endogeneity of the ∆*B*(*h*, *t*), we estimate the models in which the ∆*B*(*h*, *t*) is replaced by instrumental variable (IV) ∆B(h, 2008). As our study includes data from the same hospitals over a period of 8 years, we also applied random effects (RE) to estimate the association between the dependent DEA variable and the independent variables. The regression equation reads:

$${\mathrm{DEA}}_{ht}=\alpha +{\beta }_{1}{B}_{t}+{\beta }_{2}\Delta {B}_{ht}+{\beta }_{3}{STZ}_{h}+{\beta }_{4}{A}_{h}+{\beta }_{5}{\mathrm{HHI}}_{ht}+{{\mu }_{ht}+\varepsilon }_{ht}$$

Appendix 2 presents the results of a fixed effects model, which might be considered preferable over a random effects model because the variation across hospitals is not random and not uncorrelated with the independent variables included (as is confirmed by the Hausman test). However, the fixed effects model has the disadvantage that the instrumental variable and the hospital attractivity which are time invariant become part of the fixed effects, thus making it impossible to test our hypotheses regarding competition in the patient-hospital market.

The second modelling approach aims to circumvent the difficulties related to the asymmetries introduced by the DEA score upper bound of 1. It applies a log odds transformation of the dependent variable DEA(*h*, *t*) as mentioned above. The equation of the log odds transform linear regression with random effects reads:

$$\mathrm{ln}\left(\frac{{\mathrm{DEA}}_{(h,t)}}{1-{\mathrm{DEA}}_{(h,t)}}\right)=\alpha +{\beta }_{1}{B}_{t}+{\beta }_{2}\Delta {B}_{ht}+{\beta }_{3}{\mathrm{STZ}}_{h}+{\beta }_{4}{A}_{h}+{\beta }_{5}{\mathrm{HHI}}_{ht}+{\mu }_{ht}+{\varepsilon }_{ht}$$

Because DEA scores are bounded between 0 and 1, recoding (‘winsorizing’) all scores of 1 by an arbitrary value of 0.9999 can prevent discarding data as a result of the log odds transformation. [8]. Alternatively, we followed the approach of generalized estimation equation (GEE) with the logit link function. GEE method is an extension of the Generalised Linear Model (GLM) to model unbiased regression coefficients with a dependent variable that may not follow a normal distribution and when applied to panel data [5, 60, 82].

Finally, we applied the approach of Simar and Wilson [69] as presently more widely used and considered preferable for the reasons presented above [4]. For each of the three approaches, Appendix 1 provides results on VRS scores, instead of CRS scores, by means of a sensitivity analysis.

*Malmquist index*

To answer the second research question we used OLS to explore the association between the components of the health reform and the technical efficiency TEC(*h*,*t*) (Catch-up). For completeness, we also report the association with the Malmquist Index itself, MI(*h*,*t*), and with TECC(*h*,*t*) (Frontier Shift). For the Malmquist Index itself for instance, the linear regression equation reads:

$${\mathrm{MI}}_{ht}=\alpha +{\beta }_{1}{B}_{t}+{\beta }_{2}\Delta {B}_{ht}+{\beta }_{3}{\mathrm{STZ}}_{h}+{\beta }_{4}{A}_{h}+{\beta }_{5}{\mathrm{HHI}}_{ht}+{\varepsilon }_{ht}$$

### Data sources

All collected data included the complete set of Dutch general hospitals. As explained above, we excluded eight academic hospitals. Moreover, we excluded three specialty hospitals (eye hospital, cancer institute and orthopaedic hospital) because of their distinct case mix and cost structure. Lastly, we excluded six hospitals for which data was not available over the years 2008–2015. Including merged hospitals as outlined below, this resulted in a set of 72 hospitals out of a total of 89 Dutch hospitals.

Data were obtained from three sources. Hospital input and output data to calculate efficiency were collected from ZinData for the years 2008–2010 and from the Dutch Department of Health, Welfare and Sports (www.jaarverslagenzorg.nl) for the year 2011–2015.

The reform distinguishes two segments of Diagnosis Treatment Combinations (DTC), the Dutch variation of DRGs for which hospitals can claim reimbursement. Prices for DTC in Segment A are fixed prices by the government. Prices for DTC in Segment B are freely negotiated between hospitals and insurers. We received data on the total value of claims for each segment, per hospital, per insurer and per year from Vektis, the business intelligence centre founded by Dutch health insurers, after receiving permission from all insurers. These data enabled us to calculate the fraction of freely negotiable DTCs and insurer market power.

Data to calculate hospital market power were obtained from CBS Statline (2007–2013) and Google (2014–2015) for population size per postal code, and from Geodan for travel distances between postal codes. DEA scores were calculated by a linear programming model solved in CPLEX.

There have been 14 mergers among hospitals over the years 2008–2015 [1]. For technical reasons, most of these mergers materialized in 2015 [81]. Instead of excluding 2015 from our dataset, we considered the data for the merged hospitals to apply to each of their constituents. This allowed us to maintain a consistent sample of the largest size. We excluded three mergers from our dataset. One merger was excluded because two previously merged hospitals merged with a third hospital in the time frame of our dataset. The second merger was excluded because it was later cancelled and the third merger was excluded due to inconsistent data. We refer to Van Ineveld et al. [78] for an exploration of the relative performance of hospitals involved in mergers.

## Results

### DEA: descriptive statistics

**Fig. 2:**
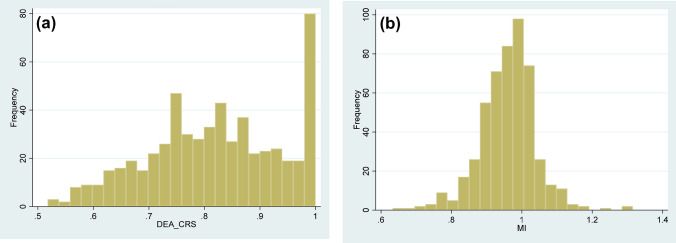
**a** Distributions of the DEA_CRS variable. **b** Distributions of the MI variable

Table 2 shows the descriptive statistics of the dependent variable *DEA* (from an input-oriented CRS model) and of the independent variables modelling the regulated competition components. Our dataset contains data from 72 hospitals for the period from 2008 to 2015. Out of 72 hospitals, 28 were STZ affiliated in 2008, and this has not changed over the study period. Appendix 3 (Fig. 2a) presents the DEA CRS distribution.

**Table 2:** Summary statistics of key variables in the regression model

Year	2008	2009	2010	2011	2012	2013	2014	2015
Hospital (*N*)	72	72	72	72	72	72	72	72
DEA								
Mean	0.83	0.84	0.83	0.83	0.83	0.81	0.80	0.79
Sd	0.11	0.11	0.12	0.12	0.12	0.13	0.12	0.13
Min	0.59	0.58	0.56	0.56	0.53	0.52	0.55	0.53
Max	1.00	1.00	1.00	1.00	1.00	1.00	1.00	1.00
*B*								
Mean	0.24	0.34	0.35	0.35	0.93	0.93	0.92	0.91
∆*B*								
Mean	0.00	0.00	0.00	0.00	0.00	0.00	0.00	0.00
Sd	0.03	0.04	0.03	0.03	0.04	0.05	0.04	0.05
Min	− 0.11	− 0.14	− 0.13	− 0.12	− 0.15	− 0.18	− 0.15	− 0.15
Max	0.06	0.08	0.06	0.06	0.05	0.06	0.05	0.06
*A*								
Mean	0.27	0.27	0.27	0.27	0.27	0.27	0.27	0.27
Sd	0.23	0.23	0.23	0.23	0.23	0.23	0.23	0.23
Min	0.07	0.07	0.07	0.07	0.07	0.07	0.07	0.07
Max	0.95	0.95	0.95	0.95	0.95	0.95	0.95	0.95
maxIMS								
Mean	0.49	0.47	0.45	0.43	0.4	0.36	0.36	0.31
Sd	0.27	0.27	0.28	0.29	0.29	0.31	0.30	0.30
Min	0.00	0.00	0.00	0.00	0.00	0.00	0.00	0.00
Max	0.89	0.77	0.76	0.77	0.76	0.77	0.76	0.76
IMS2								
Mean	0.08	0.07	0.07	0.06	0.05	0.05	0.04	0.03
Sd	0.12	0.11	0.11	0.10	0.10	0.09	0.09	0.08
Min	0.00	0.00	0.00	0.00	0.00	0.00	0.00	0.00
Max	0.36	0.36	0.34	0.34	0.33	0.32	0.28	0.27
HHI								
Mean	0.42	0.40	0.40	0.40	0.38	0.38	0.37	0.36
Sd	0.10	0.09	0.09	0.09	0.09	0.09	0.08	0.08
Min	0.22	0.21	0.21	0.21	0.21	0.21	0.21	0.21
Max	0.80	0.61	0.60	0.62	0.60	0.61	0.59	0.60

For the dependent variable, DEA, we see that after peaking at 0.84 in 2009, the average steadily decreased to 0.79 in 2015. The lowest scores are between 0.50 and 0.60 in each of the years. The average fraction of hospital revenue formed by the B segment (*B*) increased from 0.24 in 2008 to 0.93 in 2012 after which it slightly decreased again. The deviations, as captured by *ΔB* appear to have become larger over time, as witnessed by the increased standard deviation (from 0.03 to 0.05) and lower minimum values (ranging from − 0.11 to − 0.18). Deviations upwards are smaller and quite steady with a maximum of 0.06. The hospital market shares are very stable over time as hospital (location) numbers and population data are constant. The average attractiveness score *(A)* is 0.27, which can informally be interpreted as Dutch patients having on average four nearby hospitals to choose from. The insurer market shares are much more variable, which might be explained by Dutch citizens who switched between insurers and by insurers starting to actively influence the hospital choices of their customers [55].

Appendix 4 (Table 10) presents the correlation matrix between all independent variables. The control variable STZ displays a correlation of − 0.50 with Δ*B*, reflecting that STZ hospitals have lower B-Segment shares. Results without the (control) variable STZ are presented in Appendix 5 and addressed in the Discussion. The variance inflation ratio (VIF) for multicollinearity is below 1.50 (average is 1.39) for all variables, including STZ [56].

### DEA: regression results

Table 3 presents the random effects univariate regression models including each of the independent variables presented in the methods section and in Table 2. Table 3 shows that in most univariate regression models the regression coefficient is statistically significant (except in Model 4 and 7), with *B*(*t*), Δ*B*(*h*, *t*)*,* and STZ significant at level *p* < 0.01 and *A*(*h*) at the level *p* < 0.10. At 0.359 and 0.415, respectively, the univariate models with Δ*B*(*h*, *t*) and *STZ* show high levels of explained variance. Table 3 reveals that the relationship between the market share of the largest insurer per hospital and the DEA score is significant (Model 5). This model has an explained variance close to zero, and the same goes for the univariate HHI-index model which is the only other significant result (Model 8). The insurer with the largest market share per hospital is not significantly related to DEA scores (Model 7).

**Table 3:** Effects of different components of competition on DEA CRS

	Model 1	Model 2	Model 3	Model 4	Model 5	Model 6	Model 7	Model 8
*B*(*t*)	− 0.043*** (0.008)							
∆*B*(*h*, *t*)		0.860*** (0.136)						
STZ			− 0.159*** (0.018)					
*A*(*h*)				− 0.033 (0.057)				
maxIMS					0.050*** (0.018)	0.052** (0.021)		
IMS2						− 0.011 (0.052)		
I1							− 0.026 (0.075)	
I2							− 0.003 (0.062)	
I3							− 0.006 (0.067)	
I4							− 0.043 (0.069)	
I5							− 0.011 (0.072)	
HHI								0.254*** (0.069)
_cons	0.847*** (0.014)	0.820*** (0.009)	0.882*** (0.011)	0.829*** (0.020)	0.800*** (0.015)	0.800*** (0.015)	0.838*** (0.066)	0.722*** (0.030)
Obs	576	576	576	576	576	576	576	576
*R*^2^	0.012	0.359	0.415	0.005	0.004	0.005	0.032	0.054

Dependent variable: DEA_CRS. Standard errors in parentheses

***p < 0.01, **p < 0.05, *p < 0.1

The multivariate random effects (RE) model, shown in Table 4, explains more than half of the DEA score variance, and *B*(*t*)*, *Δ*B*(*h*, *t*)*,* and *STZ*, are all significant at p < 0.01. *A*(*h*) is significant at *p* < 0.1. The insurer market share coefficients are not significantly different from zero in these models. The results indicate that the increase of the B-segment is associated with a decrease in efficiency (*β* ≈ − 0.042), whereas a positive deviation from the average fraction of the B-segment is associated with higher efficiency in the same order of magnitude (*β* ≈ 1.646). STZ hospital membership is associated with a lower DEA score (*β* ≈ − 0.089). Hospitals with higher market power (i.e., facing less competition), as reflected in a higher *A*(*h*), are significantly less efficient (*β* ≈ − 0.06).

**Table 4:** Model results for the RA models with instrumental variable ∆B(*h*,2 008), the GEE model and the Stochastic Frontier Model

	(1) RE	(2) RE (IV)	(3) GEE (logit)	(4) Simar and Wilson
*B*(*t*)	− 0.042*** (0.009)	− 0.042*** (0.009)	− 0.285*** (0.079)	− 0.051*** (0.013)
∆*B*(*h*, *t*)	0.611*** (0.132)	1.646*** (0.288)	3.111** (1.290)	1.127*** (0.108)
STZ	− 0.133*** (0.017)	− 0.089*** (0.019)	− 0.907*** (0.145)	− 0.093*** (0.009)
*A*(*h*)	− 0.058 (0.036)	− 0.061* (0.033)	− 0.459 (0.309)	− 0.051*** (0.018)
HHI	0.030 (0.068)	0.027 (0.068)	0.165 (0.721)	0.002 (0.051)
_cons	0.902*** (0.030)	0.887*** (0.030)	2.201*** (0.286)	0.890*** (0.023)
/sigma				0.079*** (0.003)
Obs	576	576	576	508
*R*^2^				
Within	0.067	0.043		
Between	0.627	0.657		
Overall	0.511	0.514		

Standard errors are in parenthesis

***p < 0.01, **p < 0.05, *p < 0.1

Table 4 shows very similar results when applying the logistic regression (GEE) approach (model 3). The variables *B*(*t*), Δ*B*(*h*, *t*), are significant at *p* < 0.01 and STZ is significant at *p* < 0.05. The directions of the coefficients are also the same as the directions found with the RE model. As the interpretation of the coefficients of the logistic model are not straightforward, the marginal effects are provided in Appendix 6. The marginal effect reflects the change in the DEA score due to a unit change in one of the independent variables. It shows that a 1% increase in average B-segment corresponds to a 0.043 lower DEA score. Model 4 shows the results of the Simar and Wilson [69] approach. This analysis presents again similar results with the exception that *A*(*h*) is significant: higher hospitals attractiveness is associated with lower efficiency (*β* *≈* − 0.051).

### Technical efficiency and Malmquist index: descriptive statistics

Table 5 displays the descriptive statistics of the dependent variable TEC (catch-up) and of the related MI and TECC (frontier-shift), averaged over all hospitals per year. Over the period 2008–2015, the average TEC is quite stable, between 1.01 and 0.98. The average MI displays a substantial decline although it appears to increase again from 2013 onwards. The TEC also dropped over time, starting with scores above 1 (technological gains) but dropping to values around 0.95 from 2011–2011 onwards, which somewhat surprisingly hints at externally caused efficiency losses (as covered in the Discussion). Appendix 3 (Fig. 2b) presents the MI distribution.

**Table 5:** Summary of descriptive statistics of MI, EC and TEC

	2008–2009	2009–2010	2010–2011	2011–2012	2012–2013	2013–2014	2014–2015
MI							
Mean	1.01	1.00	0.99	0.93	0.91	0.93	0.95
SD	0.06	0.06	0.08	0.06	0.07	0.09	0.09
Min	0.88	0.73	0.81	0.74	0.71	0.69	0.63
Max	1.29	1.14	1.23	1.09	1.11	1.32	1.18
EC							
Mean	1.01	0.98	1.01	1.00	0.98	0.99	0.99
SD	0.05	0.05	0.07	0.07	0.07	0.09	0.09
Min	0.88	0.81	0.86	0.80	0.75	0.77	0.63
Max	1.23	1.11	1.29	1.14	1.19	1.45	1.21
TEC							
Mean	1.00	1.02	0.98	0.94	0.93	0.94	0.96
SD	0.02	0.04	0.04	0.03	0.03	0.03	0.03
Min	0.96	0.91	0.87	0.85	0.87	0.85	0.90
Max	1.05	1.14	1.08	1.02	1.04	0.98	1.03

### Technical efficiency and malmquist index: regression results

Table 6 shows the results of the MI, TEC and TECC OLS regression analysis. The B-segment variable *B*(*t*) is the only variable that is consistently significant. In model 1 and 3, it is very significantly and negatively associated with each of the three independent variables (*p* < 0.01) and in model 2 this relationship is significant (*p* < 0.05). We find that the B-segment increases were associated with decreases in Malmquist productivity index (MI) (*β* ≈ − 0.086), a negative technical efficiency change (EC) (*β* ≈ − 0.025) and a negative frontier shift (TEC) *(β* ≈ − 0.063). In model 1, Δ*B*(*h*, *t*) is also significantly and negatively associated with MI at the level of *p* < 0.05, but not with technical efficiency. All other independent variables do not show significant associations with the independent variables.

**Table 6:** MI regression results OLS model

	(1) MI	(2) TEC (efficiency change)	(3) TECC (frontier shift)
*B*(*t*)	− 0.086*** (0.011)	− 0.025** (0.011)	− 0.063*** (0.006)
∆*B*(*h*, *t*)	− 0.202* (0.106)	− 0.155 (0.103)	− 0.063 (0.055)
STZ	− 0.011 (0.008)	− 0.013 (0.008)	0.001 (0.004)
*A*(*h*)	− 0.013 (0.016)	− 0.006 (0.016)	− 0.008 (0.008)
HHI	− 0.026 (0.043)	− 0.011 (0.041)	− 0.016 (0.022)
_cons	1.029*** (0.019)	1.020*** (0.018)	1.011*** (0.010)
Obs	504	504	504
*R*-squared	0.113	0.017	0.196

Standard errors are in parenthesis

***p < 0.01, **p < 0.05, *p < 0.1

## Discussion

Our longitudinal analysis of the development of hospital efficiency after the introduction of managed competition in the Netherlands has revealed several noteworthy relationships between the components of managed competition and hospital efficiency over the years 2008–2015. We first summarize and reflect on the main findings.

The nationwide fraction of freely negotiated hospital services *B*(*t*) is negatively associated with relative efficiency as measured by the DEA scores. Hence, the relative efficiency has decreased as the B-segment share of hospital revenue increased over the years. To interpret this finding, it is important to recall that the DEA score is a cross-sectional score where efficiency of a hospital is compared to the efficiency of other hospitals in the same year. Hence, a decrease in average efficiency means that hospitals on average have become less efficient compared to the most efficient hospital(s) in the same year.

This finding, however, has no implication for the yearly efficiency developments of hospitals. The results presented in Table 6, however, show that the longitudinal Malmquist Index is negatively and significantly associated with *B*(*t*)*,* indicating that productivity has decreased as the B-segment grew. The decomposition of the Malmquist Index shows that hospitals’ average technical efficiency change (TEC) was also negative between 2008 – 2015 and negatively and significantly associated with B-segment, an increase in B-segment results in a negative technical efficiency change (catch-up). These results indicate that the longitudinal heath system reform did not have the intended effects on efficiency change. Instead hospitals’ relative and technical efficiency declined as free price negotiations expanded. A possible explanation is that the increase in the freely negotiable B segment has incentivized some hospitals to focus on efficiency and hence on services with high output volumes for the inputs, while others (such as the teaching hospitals) have succeeded to differentiate towards services that are less affected by competition but are provided less efficiently. It appears indeed that the entrepreneurial supply side powers have been stronger than the demand side efforts to improve efficiency through insurer purchasing practices [72, 73].

The results obtained for the fraction of freely negotiated health services per hospital *Δ(B(h,t))* appear to better align with the intended effects of the managed competition design. In all regression models presented in Table 4, Δ(*B*(*h*, *t*) is positive and significantly associated with the DEA score*,* which indicates that more efficient hospitals have relatively more B segment services. Caution is called for, however, as the Δ(*B*(*h*, *t*) might be endogeneous: higher efficiency scores might cause hospitals to enlarge their B-segment more than others, possible by being awarded with better contracts by health insurers. Our regression random effect model using an instrumental variable show that the alternative time invariant instrumental variable Δ(*B*(*h*, 2008) is also highly significant. In fact the coefficient of Δ(*B*(*h*, 2008) is larger than the coefficient of Δ(*B*(*h*, *t*), which might be impacted by correlation with the control variable STZ. In the longitudinal regression model, however, Δ(*B*(*h*, *t*) is not significantly related to technical efficiency change, indicating that higher B-segment shares are not associated with efficiency improvement. This would confirm that the hospitals which had higher B-segment shares in 2008 have mainly consolidated their efficiency differences until 2015.

Our initial motivation for introducing the binary variable *STZ* has been to control for case mix. Our results reveal that STZ membership is significantly and substantially negatively associated with efficiency. STZ members on average have a 0.13 lower DEA score on an average overall DEA score ranging from 0.83 to 0.79 over the years (0.09 lower in the models with the instrumental variable). The results in Appendix 3 show that removing the control variable *STZ* from the multivariate models reduces the explained variance by around 0.13 (Appendix 5) to around 0.39. The coefficient for *STZ* is, however, not statistically significant in the variable returns to scale (VRS) DEA results presented in Appendix 1. Together these findings suggest a strong relationship between case mix and volume (Appendix 1). The STZ hospitals typically provide more complex health services and have larger volumes at the same time. Larger volumes and sizes have been associated with diseconomies of scale as measured in number of beds) [37]. Moreover, previous research shows that the optimal scale is between 200 and 400 beds [28]. The negative effect of (dis)economies of scale on efficiency can, therefore, be stronger than the case-mix effect for STZ members. Further research is called for to disentangle the relationship between volume, case-mix, and economies of scale.

Results from the multivariate regression models with hospital market share *A*(*h*) are ambiguous. In the RE model and the GEE model, the association between *A*(*h*) and DEA_CRS is not significant. However, the RE model with instrumental variable and the Simar and Wilson 2 model confirm the hypothesis that hospitals are more efficient as the patients within 30 min travel distance of the local hospital have more alternative hospitals within 30 min travel distance. Increased competition in the market between hospitals and patients is associated with higher efficiency. The effect is highly significant in the Simar and Wilson model, but the effect size is small (0.05 efficiency loss from infinite competition to a monopolist). It should be noted that the scores of the most efficient hospitals (with a DEA score of 1) are excluded in the Simar and Wilson model, as assumed artifacts of finite sample bias [4]. Interestingly, there is no significant relationship of hospital market share *A*(*h*) with technical efficiency change, suggesting that competition density did not affect technical efficiency development over time. These results are broadly in line with findings on quality improvement obtained for 2006–2008 by Roos et al. [64]. However, they struggle with robustness, a problem which may well be resolved by the methodological advancements to model hospital market power presented in this study.

All variables modelling insurer competition and insurer purchasing practices are non-significant in the multivariate analysis and explain at most 0.01 of the variation in the univariate models. Hence, the results indicate that competition between insurers has no relationship with efficiency. This is further confirmed by the finding that none of the insurers is significantly associated with hospital efficiency for the hospitals for which they act as the main insurer: differences in purchasing practices have not translated into significant efficiency differences. Our unique and detailed data set has served to reject the hypothesis that competition on the market between insurers and hospitals has improved efficiency in The Netherlands. This is confirmed by the insignificance of relationships with VRS DEA scores (Appendix 1) and Technical Efficiency. A possible explanation is that insurers have focused on the quality of care rather than efficiency. Unfortunately, there is no relevant (hospital-wide) quality of care data that have been consistently reported over the sample period [19, 78]. Hence this remains a topic for future research. Another possible explanation is that Dutch health insurers have hardly practiced selective contracting, which has been suggested to be caused by a lack of credible commitment [70, 13].

A fixed effects model might be considered preferable over a random effects model as the variation across hospitals is not random and not uncorrelated with the independent variables included in the model. However, the fixed effect absorbs the time-invariant variables *STZ* and *A*(*h*). Hence a fixed effects model cannot test the hypothesis regarding competition on the patient-hospital market. Moreover, any controlling for case mix as done by the STZ variables is then consumed by the fixed effect. Nevertheless, we mention that the fixed effects results provided in Appendix 6 provide results comparable to the random effects model for the other variables, as further confirms the robustness of the results.

## Limitations

A limitation of our study is that we have not controlled for government interventions in the regulations. For instance, the basis of the reimbursement model changed considerably in 2012 [24]. Moreover, an agreement between stakeholders limited total healthcare expenditures growth to 2.5% annually in the period 2012–2015 [3]. Other limitations are formed by the relatively crude measure introduced to model case mix (STZ variable), the lack of consistently reported hospital-wide quality data to include in the model, and the lack of an input variable to model the resource capital—despite extensive mandatory financial reporting.

## Conclusions

Despite the limitations, the data from all non-academic hospitals, all insurers, and the value of care contracted between each pair of insurers and hospitals over a period of 8 years, combined with demographic and geographic data provide an extensive data set. Together with the methodological advancements in the definition of independent variables, these data have enabled to present insightful new results on the effectiveness of the components of the Dutch health reform. Our results indicate that competition for patients and the relative fraction of care for which prices can be freely negotiated promote hospital efficiency. Interestingly and perhaps most strikingly, the steady nationwide increase of the fraction of health services contracted in competition appears to be negatively associated with efficiency. This applies to both the relative efficiency position of hospitals and the technical efficiency change of hospitals over time. Our study did not reveal any other longitudinal effects, nor did it find any significant results for insurer bargaining power or practices.

Our study contributes to the ongoing debate whether competition in healthcare is good or bad. In the Netherlands, the Covid-19 pandemic has accelerated this debate. Stakeholders argue that health systems based on the principles of managed competition struggled to control and address Covid-19 and that cooperation between providers with the support of local and national government is preferable over competition. The pandemic has not only revealed the fragility of health systems, it also put the spotlight on cooperative paradigms. For The Netherlands, our study reveals little evidence in support of a competitive model.

## Data Availability

The Erasmus University Rotterdam has access to the research data. Due to the confidentiality of data, the data are not available to third parties.

## Appendix 1: Variable returns to scale (VRS) DEA analysis

See Tables 7 and 8.

**Table 7:** Effects of different components of competition on DEA_VRS

	Model 1	Model 2	Model 3	Model 4	Model 5	Model 6	Model 7	Model 8
*B*(*t*)	− 0.027*** (0.008)							
∆*B*(*h*, *t*)		0.325** (0.130)						
STZ			− 0.035** (0.017)					
*A*(*h*)				− 0.034 (0.038)				
maxIMS					0.004 (0.017)	− 0.000 (0.019)		
IMS2						0.023 (0.049)		
I1							− 0.004 (0.069)	
I2							0.012 (0.063)	
I3							0.004 (0.066)	
I4							− 0.023 (0.066)	
I5							0.006 (0.067)	
HHI								0.033
								(0.063)
_cons	0.931*** (0.010)	0.914*** (0.008)	0.928*** (0.011)	0.924*** (0.014)	0.913*** (0.011)	0.913*** (0.011)	0.914*** (0.064)	0.901*** (0.026)
/sigma								
Obs	576	576	576	576	576	576	576	576
Overall *R*^2^	0.007	0.066	0.034	0.008	0.001	0.006	0.034	0.002

Dependent variable: DEA_VRS. Standard errors in parentheses

****p* < 0.01, ***p* < 0.05, **p* < 0.1

**Table 8:** Comparing DEA VRS model results for the random effect models with instrumental variable ∆*B*(*h*, 2008), the generalized estimation equation model and Simar and Wilson model

	(1) RE	(2) RE(IV)	(3) GEE (logit)	(4) Simar a Wilson
*B*(*t*)	− 0.030*** (0.009)	− 0.030*** (0.009)	− 0.384** (0.151)	− 0.070*** (0.022)
∆*B*(*h*, *t*)	0.266* (0.136)	0.881*** (0.318)	2.641 (2.195)	0.962*** (0.183)
STZ	− 0.026 (0.018)	0.000 (0.021)	− 0.342 (0.242)	− 0.017 (0.016)
*A*(*h*)	− 0.027 (0.038)	− 0.029 (0.037)	− 0.429 (0.533)	− 0.059* (0.033)
HHI	− 0.068 (0.071)	− 0.071 (0.071)	− 0.921 (0.940)	− 0.116 (0.086)
_cons	0.977*** (0.032)	0.968*** (0.031)	3.251*** (0.322)	1.023*** (0.041)
/sigma				0.100*** (0.006)
Obs	576	576	576	390
*R* ^2^				
Within	0.024	0.013		
Between	0.117	0.128		
Overall	0.083	0.083		

Standard errors are in parenthesis

****p* < 0.01, ***p* < 0.05, **p* < 0.1

## Appendix 2

See Table 9.

**Table 9:** Effects of different components of competition on DEA_CRS fixed effects model

	(1) FE	(2) FE (IV)
*B*(*t*)	− 0.032*** (0.009)	− 0.032*** (0.011)
∆*B*(*h*, *t*)	0.465*** (0.154)	− 1.654** (0.816)
STZ		
*A*(*h*)	7.182*** (2.415)	
HHI	0.079 (0.089)	0.225* (0.116)
_cons	− 1.159* (0.663)	0.753*** (0.049)
Obs	576	576
*R* ^2^		
Within	0.088	
Between	0.005	0.431
Overall	0.041	0.277

Standard errors are in parenthesis

****p* < 0.01, ***p* < 0.05, **p* < 0.1

## Appendix 3

See Fig. 2.

## Appendix 4

See Table 10

**Table 10:** Correlation matrix

Variables	(1)	(2)	(3)	(4)	(5)	(6)	(7)	(8)	(9)	(10)	(11)	(12)
(1) *B*(*t*)	1.000											
(2) ∆*B*(*h*, *t*)	− 0.000	1.000										
(3) * A*(*h*)	− 0.001	0.040	1.000									
(4) STZ	0.000	− 0.535	− 0.043	1.000								
(5) maxIMS	− 0.177	− 0.013	0.383	− 0.192	1.000							
(6) IMS2	− 0.130	− 0.110	0.178	0.010	0.338	1.000						
(7) I1	0.000	0.037	0.267	− 0.043	0.085	− 0.205	1.000					
(8) I2	0.013	− 0.048	− 0.206	− 0.076	0.224	− 0.205	− 0.278	1.000				
(9) I3	− 0.023	0.178	− 0.122	0.073	− 0.160	− 0.133	− 0.122	− 0.236	1.000			
(10) I4	− 0.004	− 0.066	− 0.061	0.164	− 0.153	0.299	− 0.209	− 0.407	− 0.178	1.000		
(11) I5	-0.000	− 0.033	0.179	− 0.104	− 0.056	0.196	− 0.186	− 0.361	− 0.158	− 0.272	1.000	
(12) HHI	− 0.175	0.006	0.426	− 0.196	0.807	0.238	0.056	0.174	− 0.233	− 0.039	− 0.034	1.000

## Appendix 5

See Tables 11 and 12.

**Table 11:** Effects of different components of competition on DEA_CRS excluding variable STZ

	Model 1	Model 2	Model 3	Model 4	Model 5	Model 6	Model 7
*B*(*t*)	− 0.043*** (0.008)						
∆*B*(*h*, *t*)		0.860*** (0.136)					
*A*(*h*)			− 0.033 (0.057)				
maxIMS				0.050*** (0.018)	0.052** (0.021)		
IMS2					− 0.011 (0.052)		
I1						− 0.026 (0.075)	
I2						− 0.003 (0.062)	
I3						− 0.006 (0.067)	
I4						− 0.043 (0.069)	
I5						− 0.011 (0.072)	
HHI							0.254*** (0.069)
_cons	0.847*** (0.014)	0.820*** (0.009)	0.829*** (0.020)	0.800*** (0.015)	0.800*** (0.015)	0.838*** (0.066)	0.722*** (0.030)
Obs	576	576	576	576	576	576	576
*R* ^2^	0.012	0.359	0.005	0.004	0.005	0.032	0.005

Standard errors are in parenthesis

****p* < 0.01, ***p* < 0.05, **p* < 0.1

**Table 12:** Comparing DEA_CRS without STZ variable model results for the random effect models with instrumental variable ∆B(h,2008), the Generalized Estimation Equation model and Simar and Wilson model

	(1) RE	(2) RE(IV)	(3) GEE (logit)	(4) Simar and Wilson
*B*(*t*)	− 0.038*** (0.009)	− 0.040*** (0.010)	− 0.255*** (0.079)	− 0.044*** (0.014)
∆*B*(*h*, *t*)	0.836*** (0.133)	2.253*** (0.279)	3.887*** (1.282)	1.734*** (0.100)
*A*(*h*)	− 0.058 (0.042)	− 0.061 (0.041)	− 0.491 (0.363)	− 0.066*** (0.021)
HHI	0.094 (0.073)	0.057 (0.077)	0.579 (0.944)	0.137** (0.054)
_cons	0.824*** (0.031)	0.840*** (0.032)	1.613*** (0.347)	0.799*** (0.022)
/sigma				0.088*** (0.003)
Obs	576	576	576	508
*R* ^2^				
Within	0.062	0.062		
Between	0.524	0.518		
Overall	0.385	0.371		

Standard errors are in parenthesis

****p* < 0.01, ***p* < 0.05, **p* < 0.1

## Appendix 6

See Table 13.

**Table 13:** Marginal effects logistic regression model

	d*y*/d*x*	Std. err	*P* > *z*	[95% CI]	
*B*(*t*)	− 0.040	0.011	0.000	− 0.061	− 0.018
∆*B*(*h*, *t*)	0.432	0.181	0.017	0.077	0.787
STZ	− 0.135	0.020	0.000	− 0.175	− 0.095
*A*(*h*)	− 0.064	0.042	0.130	− 0.146	0.019
HHI	0.023	0.100	0.819	− 0.173	0.219

## Appendix 7

See Table 14.

**Table 14:** Correlation matrix

Variables	FTE	OpExp	Adm	Dayc	FirstOutpV
FTE	1.000				
OpExp	0.924	1.000			
Adm	0.956	0.867	1.000		
Dayc	0.852	0.758	0.876	1.000	
FirstOutpV	0.941	0.838	0.952	0.875	1.000
